# Independent Medical Journals and Official Organs

**Published:** 1913-11

**Authors:** 


### BUFFALO MEDICAL JOURNAL

**A Monthly Review of Medicine and Surgery**

EDITOR AND PUBLISHER DR. A. L. BENEDICT, 228 Summer St., corner of Elmwood Ave., Buffalo. (Address for all communications. Please make personal and telephone calls before 1 P. M.)

Third, in age, in the western continent. Independent but supports professional organization. News limited mainly to western N. Y. and Penn.

Subscription $2 00 a year; $3.00 for two years in advance. Changes and errors in address or failure or duplication of delivery, should be reported immediately.

YEARLY VOLUME 69 NOVEMBER, 1913 No. 4

### Independent Medical Journals and Official Organs

The profession of this country has what we consider the largest and best organ in the world as well as a considerable number of lesser ones, some very good, some rather poor, and with no regular relation between the excellence of the organ and the size and prosperity of the congregation. There has been much complaint about these various organs, both by individual members of the congregations and by those who are more or less interested in other instruments. The complaints may be reduced to two general categories. First, the organs are played by regular organists and the hymns are selected by the minister and deacons, sometimes with the advice of prominent pillars in the congregation. This fact arouses some jealousy and a good deal of honest difference of opinion as to the selection of tunes. In our opinion, this restriction is inevitable, and we approve heartily of the tunes played, although, of course, there are times when we might have a personal preference for a different selection, obiect to the repetition of a favorite, or wish that there might be a possibility of suiting the wishes of some humble member of the congregation who has his own favorites.

Secondly, there are abundant opportunities for criticism of the technic of the organist. Sometimes these criticisms are obviously captious, sometimes they are sincere. Sometimes we agree with them and sometimes we do not. Occasionally we may think it worth while to make these criticisms in a frank and friendly spirit; more often we approve of the policy of the pioneer western church which posted a sign over the organist: "Don't shoot the organist; he is doing the best he can." If he strikes a false note once in a while, or if the music is too Wagnerian, or if the expression and tempo do not correspond with our own interpretation, or if the congregation or a soloist are drowned out by the organ, it is well to remember that no one is perfect and no one can please everybody all the time.

On the other hand (and let it be understood that this opinion is a personal one, long antedating any editorial interest) we do not believe that the profession should be or wants to be restricted to organ music. There is need for other instruments of independent nature. Some of these instruments are more powerful and of better tone than many of the organs. We think that we have a pretty fair one. Anyone who has subscribed to it can play any tune he likes on it, except the Mocking Bird and the Anvil Chorus. There is no order of precedence and no rule against encores, except such as ordinary courtesy and common sense suggests, when several want to play at the same time. There is not even any objection to its use by those outside the congregation, excepting the same qualification. Rather frequently, distinguished composers favor us with selections.

In closing, we wish only to say that we have no sympathy whatever with those interested in independent instruments who try to cut slits in the bellows or to throw sand in the motors of the organs.

